# Exploring effectiveness of CBT in obese patients with binge eating disorder: personality functioning is associated with clinically significant change

**DOI:** 10.1186/s12888-023-04626-x

**Published:** 2023-03-06

**Authors:** Laura van Riel, Elske van den Berg, Marike Polak, Marjolein Geerts, Jaap Peen, Theo Ingenhoven, Jack Dekker

**Affiliations:** 1 Centre for Eating Disorders and Obesity, Novarum, Arkin Institute of Mental Health, Amsterdam, The Netherlands; 2 Department of Psychology, Education & Child Studies (DPECS), Erasmus University Rotterdam, Rotterdam, The Netherlands; 3 Department of Research, Arkin Institute of Mental Health, Amsterdam, The Netherlands; 4 Centre for Personality Disorders, NPI, Arkin Institute of Mental Health, Amsterdam, The Netherlands

**Keywords:** Obesity, Binge eating disorder, Personality traits, Personality functioning, Psychological assessment, Temperament and character inventory, Developmental Profile Inventory, Cognitive behavioral treatment, Clinical significant change

## Abstract

**Background:**

Binge eating disorder (BED), as the most prevalent eating disorder, is strongly related to obesity and other somatic and psychiatric morbidity. Despite evidence-based treatments a considerable number of BED patients fail to recover. There is preliminary evidence for the association between psychodynamic personality functioning and personality traits on treatment outcome. However, research is limited and results are still contradictory. Identifying variables associated with treatment outcome could improve treatment programs. The aim of the study was to explore whether personality functioning or personality traits are associated with Cognitive Behavioral Therapy (CBT) outcome in obese female patients with BED or subthreshold BED.

**Methods:**

Eating disorder symptoms and clinical variables were assessed in 168 obese female patients with DSM-5 BED or subthreshold BED, referred to a 6-month outpatient CBT program in a pre-post measurement design. Personality functioning was assessed by the Developmental Profile Inventory (DPI), personality traits by the Temperament and Character Inventory (TCI). Treatment outcome was assessed by the Eating Disorder Examination-Questionnaire (EDE-Q) global score and self-reported binge eating frequency. According to the criteria of clinical significance, 140 treatment completers were categorized in four outcome groups (recovered, improved, unchanged, deteriorated).

**Results:**

EDE-Q global scores, self-reported binge eating frequency and BMI significantly decreased during CBT, where 44.3% of patients showed clinically significant change in EDE-Q global score. Treatment outcome groups showed significant overall differences on the DPI Resistance and Dependence scales and the aggregated ‘neurotic’ scale. Significant overall differences were found between groups on TCI Harm avoidance, although post hoc t-tests were non-significant. Furthermore, multiple logistic regression analysis, controlling for mild to moderate depressive disorder and TCI harm avoidance showed that ‘neurotic’ personality functioning was a significant negative predictor of clinically significant change.

**Conclusion:**

Maladaptive (‘neurotic’) personality functioning is significantly associated with a less favorable outcome after CBT in patients with binge eating. Moreover, ‘neurotic’ personality functioning is a predictor of clinically significant change. Assessment of personality functioning and personality traits could support indication for more specified or augmented care, tailored towards the patients’ individual strengths and vulnerabilities.

**Trial registration:**

This study protocol was retrospectively evaluated and approved on 16-06-2022 by the Medical Ethical Review Committee (METC) of the Amsterdam Medical Centre (AMC). Reference number W22_219#22.271.

## Background

With a 0.9% lifetime prevalence Binge Eating Disorder (BED) is the most common eating disorder in adults in the general population [1]. In DSM-5 BED is characterized by recurrent, weekly episodes of uncontrollable overeating and significant distress, without compensatory behaviors as present in bulimia nervosa [2]. In ‘subthreshold BED’ all of the criteria for BED are met except that patients binge, on average, less than once a week or for less than 3 months. BED is significantly associated with obesity, major comorbid psychiatric disorders, numerous medical disorders [3–9] and significant psychosocial impairments [4, 6, 9]. Studies on efficacy showed that both cognitive behavioral therapy (CBT) and interpersonal psychotherapy (IPT), have short- and long-term benefits for BED [11]. However still about half of the patients fail to fully respond [4, 12–17]. Increasing our understanding of variables associated with treatment outcome can contribute to the development of more effective, personalized, therapy programs.

With this focus in mind, the complex relationship between BED and personality pathology has been a subject in research for years and different models provide complementary perspectives. The interpersonal model of BED [18] implies that impaired interpersonal functioning reinforces low self-esteem and negative affect, which in turn triggers binge eating as a dysfunctional strategy to cope with negative feelings [19–23]. A recent study confirms that patients with BED report higher levels of interpersonal problems, in particular greater submissiveness, as compared to both obese people without BED and normal weight controls [24]. In addition, patients with more severe eating pathology reported lower global self-esteem [25]. Attachment theory [26] takes a developmental perspective regarding eating disorder symptoms, positing that repeated interactions based on attachment behaviors with caregivers result in internal working models of the self and other and specific adult attachment styles [27]. Understanding binge eating from the perspective of attachment theory is consistent with the interpersonal model of binge eating. Within this frame of reference interpersonal problems, resulting from insecure attachment, lead to negative affect and increased use of maladaptive coping which in turn may trigger binge eating in BED [28]. Recent studies confirm that emotion dysregulation and maladaptive coping mediate the relationship between attachment anxiety and binge eating [29–33].

This integrative perspective is in line with the current hybrid DSM-5 Alternative Model of Personality Disorders (AMPD) in which psychodynamically informed levels of personality functioning -as reflected by concepts of self and interpersonal functioning- are combined with personality traits [2]. A recent study exploring personality functioning and personality traits in patients with BED showed that patients with BED or subthreshold BED presented more maladaptive and less adaptive psychodynamic personality functioning as well as impaired personality traits, as compared to obese and normal weight community controls [34].

In addition to studies investigating the associations between BED and personality pathology there is some evidence for the predictive value of low self-esteem [35, 36], interpersonal problems [37, 38] or pathological personality traits [39–41] in BED treatment outcome. However, the amount of studies regarding the associations between personality functioning as well as concomitant personality traits on BED treatment outcome is very limited. Yet, extending our knowledge about this relationship could contribute to selection of those patients who could benefit from specific psychotherapeutic treatment offers or a more personalized approach based on individual strengths and vulnerabilities.

The aim of the current study was to evaluate the outcome of BED treatment using the criteria of clinical significance. In addition, we aimed to explore the associations between CBT treatment outcome and impairments in personality functioning as well as pathological personality traits in female obese patients with BED or subthreshold BED. We hypothesize that impaired personality functioning and impaired personality traits are associated with a less favorable treatment outcome in this patients’ population with binge eating pathology regarding eating disorder behaviors and cognitions and specifically binge eating frequency.

## Methods

### Participants

The study included 168 obese women (BMI ≥ 30 kg/m^2^) with BED or subthreshold BED, aged 18–68 years (*M* = 41, *SD* = 12.6), who were referred between 2014 and 2017 for CBT to Novarum, a specialist treatment centre for obesity and eating disorders in Amsterdam, the Netherlands.

Diagnostic assessment included an initial telephone screening on eating disorder symptoms and other psychiatric symptoms and, if applicable, retrievement of information about former psychological treatments. Subsequently, a clinical interview by either a licensed and trained psychologist or psychiatrist was conducted, in which the presence of BED or subthreshold BED was determined, as well as relevant psychiatric comorbidity. The assessment for eating disorders included a semi-structured diagnostic interview based on the SCID-I eating disorder module as part of the Structured Clinical Interview for DSM-IV, Patient Edition (SCID-P) [42] including specific questions regarding criteria for binge eating as established in the Eating Disorder Examination (EDE) interview [43]. The diagnostic formulation and proposed treatment options were then confirmed by a multidisciplinary team. As DSM-5 was introduced in the Netherlands on January 2017, the former DSM-IV diagnosis of all participants were revised (by the psychiatrist of the research team) using the DSM-5 criteria for BED and subthreshold BED (as described by the DSM-5 category Other Specified Feeding and Eating Disorders (OSFED)).

Patients engaging in compensatory bulimic behaviors like vomiting or laxative misuse were not eligible for this study, nor were patients who were currently in concurrent treatment for BED or weight loss programs. Other exclusion criteria were severe comorbid psychiatric disorders (e.g. psychotic disorders, severe mood disorders, suicidality or substance use disorders), mental retardation and current pregnancy.

A total of 168 patients participated in the study, 156 of which completed their treatment program. 12 patients dropped out of treatment because of current life events, patient’s feeling that either the therapy did not meet their expectation in relief from complaints or the unilateral belief that she had already improved sufficiently. In some cases, the therapist thought a comorbid disorder required attention first. Of the 156 treatment completers, 7 patients were excluded from analysis due to missing data on one of the primary outcome variables, the EDE-Q global score or EDE-Q self-reported binge eating frequency. Remaining patients with normative EDE-Q global scores both at admission and discharge (*n* = 9) were not included in the analyses since improvement for this group was not applicable, leaving 140 patients for further analyses.

This study protocol was approved by the Medical Ethical Review Committee (METC) of the Amsterdam Medical Centre (AMC). All participants gave informed written consent before enrolment and received 15 euro compensation for their willing participation.

### Treatment program

The treatment program was based on the principles of CBT-E, Cognitive Behavioral Therapy Enhanced for eating disorders [44, 45]. Patients were offered 20 sessions of CBT once a week, either in a group (maximum nine patients) or individual. 86 patients (61.4%) received the group treatment, 54 patients (38.6%) the individual treatment. Weekly psychomotor therapy was added to the group program. The maximum absence allowed was 3 out of 20 sessions. If the client missed 4 or more sessions, they were regarded as treatment non-completers. Alongside these main sessions, all patients attended at least one additional informative group meeting (90 min) with their partner or a close relative. The main objective of this meeting was to enhance mutual understanding and support during the process of change. The main goal of the treatment program was to regain control over binge eating, establish a regular eating pattern, develop a more realistic body image, decrease body shape dissatisfaction and diminish the influence of shape and weight on self-esteem. Sessions consisted of discussing daily self-monitoring of eating behaviors, psycho-education and cognitive therapy. In addition, weight was monitored weekly. Weight loss was not the primary incentive of this treatment, but prevention of weight gain was. The group treatment had a half-open group format: new patients could enter every 10th week. All sessions were led by a CBT trained psychologist or psychotherapist.

### Measures

Prior to the treatment participants completed a set of self-rating questionnaires to assess eating disorder pathology, personality functioning and personality traits. Eating disorder pathology was also administered post-treatment.

The **Eating Disorder Examination Questionnaire (EDE-Q)** [46] is a 36-item self-report based on the Eating Disorder Examination (EDE) [43]. The EDE-Q generates frequency ratings for key eating disorder behaviors (binge-eating, self-induced vomiting, laxative misuse, and excessive exercise), dietary restraint, and concerns about weight, shape and eating. Higher scores on the EDE-Q are indicative of more severe eating disorder pathology. The self-reported binge eating frequency was measured by the EDE-Q. A global score was calculated by summing up and averaging all attitudinal items, so that each item has equal weight [47]. Psychometric analyses showed the EDE-Q global score to be moderately accurate in discriminating obese individuals with BED from those without BED [47]. The EDE-Q presents strong psychometric properties in terms of validity, internal consistency and test-retest reliability [47–50]. The validated Dutch version of EDE-Q was used in the current study [51].

The **Temperament and Character Inventory (TCI)** is a 240-item self-report for personality traits based on Cloninger’s psychobiological model [52, 53]. The TCI includes four dimensional temperament scales (‘Novelty seeking’, ‘Harm avoidance’, ‘Reward dependence’ and ‘Persistence’) and three character scales (‘Self-directedness’, ‘Cooperativeness’ and ‘Self-transcendence’). The TCI, as composed of 240 true/false items, has proven good internal consistency and test-retest reliability[54]. The validated Dutch version was used in the current study [55].

The **Developmental Profile Inventory (DPI)** [56] identifies levels of personality functioning, by the psychodynamic and behavioral patterns of an individual’s current functioning. This self-report questionnaire, based on the framework of the Developmental Profile [57, 58, 59], is organized hierarchically by nine levels of psychodynamic personality functioning. The DPI offers a strength-weakness analysis that can be helpful for meaningful case formulation, indication for psychotherapeutic treatment and identifying the most relevant psychodynamic focus during psychotherapeutic treatment. The 108 items (statements which the patient describes as being more or less applicable to him/her on a four-point Likert scale) refer to psychodynamic patterns in three domains (Self, Interpersonal Functioning and Problem-Solving Strategies) and generate scores over the nine subsequent hierarchically-ordered developmental levels (three ‘Adaptive’ levels: Maturity/Generativity, Solidarity, Individuation; three ‘Neurotic’ levels: Rivalry, Resistance, Dependence; and three ‘Primitive” levels: Egocentricity, Fragmentation, Lack of Structure). The DPI has shown adequate psychometric properties in terms of reliability and validity [60]. Dutch and English versions of the manualized DPI are available (https://www.developmental-profile.nl).

Additionally, all participants were measured bare-footed with medical scales and a stadiometer, through which BMI (kg/m2) was calculated. In addition, all patients were systematically assessed with respect to sociodemographic characteristics (age, gender, marital status, and educational level).

### Statistical analysis

In order to examine the associations between treatment outcome and personality functioning or personality traits we employed the concept of clinically significant change as proposed by Jacobson and Truax [61, 62]. Clinically significant change is defined as improvement after treatment that has taken the patient from a score typical of a clinical population to a score typical of a normal functioning population [63]. To qualify as clinically significantly improved the patient has to (a) show a reliable change (*statistically* significant improvement) and (b) cross the cut-off point for a *clinically* significant change [62]. First, a reliable change index (RCI) was calculated for the EDE-Q global score, reflecting what is considered a statistically significant and reliable change. In line with Dingemans [64], based on the method as described by Hiller [65] and clinical and general population normative data as provided by Aardoom [47], we computed an RCI of 0.79 for the EDE-Q global scores. Next, a clinical cut-off point (CO) was determined based on Dutch normative data provided by Aardoom [47], where we computed an estimated CO of 2.17 for the EDE-Q global scores. By combining the EDE-Q global score RCI (0.79) and clinical CO (2.17) four treatment outcome groups based on their EDE-Q global scores were determined:

1. Recovered: patients with both statistically significant improvement (RCI ≥ 0.79) and clinically significant recovery (CO < 2.17; i.e., symptoms within the range of the normal population sample after therapy).
2. Reliably improved: statistically significant and reliable improvement (RCI ≥ 0.79) between pre- and post-measurement, but not recovered.
3. Unchanged: patients without statistically reliable individual improvement (RCI < 0.79).
4. Deteriorated: statistically significant worsening (RCI ≤ − 0.79) of patients.

To investigate differences between pre- and post-measurement for the different treatment outcome groups, paired t-tests were computed.

Subsequently, we analyzed the associations between personality functioning and treatment outcome as well as the associations between personality traits and treatment outcome, by analyzing differences between the outcome groups on personality functioning (DPI) variables and personality traits (TCI) with separate ANOVAs and post hoc t-tests with Bonferroni corrected p-values.

As a final step, we conducted logistic regression analysis to determine the independent contributors to the odds of recovery after treatment in the whole sample. Recovery (i.e. clinically significant change) is defined by the different outcome groups (1 = recovered, 0 = not recovered). The possible predictors of recovery, which were statistically significant in single logistic regression analysis, were entered as predictors in a multiple logistic regression model (enter). Predictors that were tested as covariates were age, educational level (dummy variables), binge eating episodes, BMI, BED vs. subthreshold BED diagnosis, type of treatment (group or individual), and co-morbid DSM-5 psychiatric disorders with prevalence in our sample suited for logistic regression analysis (i.e., expected frequencies > 5), namely mild to moderate depressive disorder (present, absent) and personality disorder (present, absent). Remaining predictors were the different DPI levels of personality functioning and the various subscales of the TCI. Odds ratios (OR) and 95% confidence intervals (95% CI) were calculated for predictors. For the multiple logistic regression model a receiver operating characteristic (ROC) curve was plotted to determine the predictive value of the model. Furthermore, Nagelkerke *R*^2^ was reported as an indication of explained variance. In the multiple regression model, predictors were standardized to enhance comparison between predictors in terms of relative importance.

All analyses were executed in IBM SPSS Statistics (Version 28) with a significance level of *p* < .05. Where parametric testing was used, distribution of variables was assessed. In case skewness or kurtosis devided by its standard error exceeded the cut-off of 1.96, a non-parametric counterpart of the test was conducted to confirm parametric results [66].

## Results

### Study sample

Mean age at baseline was 41 years (*SD* = 12.6, range 18–68) years. Mean pretreatment BMI was 39.25 kg/m2 (*SD* = 5.98, range 30.11–56.70 kg/m2). 10% completed elementary school or lower vocational school, 42.9% high school or vocational school and 47.1% (under) graduate school. 89 (63.3%) participants were diagnosed with BED, 51 (36.4%) with subthreshold BED.

Prevalence of co-morbid DSM-5 psychiatric disorders was as follows: 14.3% for mild to moderate major depressive disorder, 2.1% for anxiety disorder, 2.1% for ADHD, 3.6% for PTSD, 2.1% for other DSM-5 symptom disorders. 11.4% met the criteria for personality disorders according to DSM-5 (Section II): borderline personality disorder 3.6%, obsessive compulsive personality disorder 1.4%, avoidant personality disorder 2.1% and other specified personality disorder 4.3%.

### Baseline differences between patients with BED and those with subthreshold BED

Pre-treatment, patients with BED (*n* = 89) did not differ significantly from those with subthreshold BED (*n* = 51) with respect to age, educational level and BMI. Patients with BED were relatively more often assigned to group therapy (68.5%) than to individual therapy (31.5%) as compared to patients with subthreshold BED (49% vs. 51%). In line with the clinical criteria for distinguishing between BED and subthreshold BED, patients with BED scored significantly higher with respect to the EDE-Q global score and binge eating frequencies. Furthermore, patients with BED scored significantly higher on the DPI levels of personality functioning Lack of Structure, Fragmentation, Egocentricity and Rivalry and the aggregated developmental levels, ‘Primitive’, ‘Neurotic’ and overall ‘Maladaptive’ functioning than subthreshold BED patients. In addition, patients with BED scored significantly higher with respect to the TCI personality trait Reward dependency.

As patients with BED showed a more maladaptive personality functioning profile, we investigated BED as potential covariate in the analysis of treatment outcome and in the prediction of treatment outcome.

### Baseline differences between treatment completers and treatment drop-outs

Treatment completers (*n* = 140) did not differ significantly from treatment non-completers (*n* = 12) with respect to age, educational level, EDE-Q global score, binge eating frequencies, BMI, DPI levels of personality functioning and type of treatment (group or individual). However, some significant differences in TCI personality traits existed: treatment drop-outs had significantly higher scores on Harm avoidance (d = 0.61) and Novelty seeking (d = 0.69) than patients who completed treatment. For the following part, only analyses of treatment completers will be considered.

### Improvement after treatment

Table 1 shows overall differences pre-and post-treatment with respect to EDE-Q global scores, EDE-Q self-reported binge eating frequency and BMI. Paired t-tests showed that patients’ EDE-Q global scores as well as their self-reported binge eating frequency significantly decreased during treatment (*p* < .001) with a large effect size. Notably, also patients’ BMI scores significantly decreased during treatment (*p* < .001), however with a small effect size.

**Table 1:** Pretreatment and post-treatment means (SD) for treatment completers (*n* = 140)

		Pretreatment scores	Post-treatment scores									
		M (SD)	M (SD)	Test statistics	*p*	Hedges’s g_av_						
EDE-Q global score		3.64 (0.80)	2.25 (1.16)	t(139) = 13.75	*p* < .001	1.40						
EDE-Q OBE scale scores		7.12 (9.34)	1.49 (2.80)	t(137) = 7.425	*p* < .001	0.80						
BMI		39.90 (5.97)	38.63 (5.80)	t(138) = 4.35	*p* < .001	0.10						

Notes: EDE-Q, Eating Disorder Examination Questionnaire; BMI, body mass index; p = p value. Hedges’s g_av_ effect size for change scores: 0.20 = small; 0.50 = medium; 0.8 = large [67]. Given positive skewness of distributions of difference scores for the above three outcome variables (skewness/SE > 1.96), Wilcoxon tests were computed to confirm parametric results. Non-parametric test statistics for the EDE-Q global scores, binge eating frequency and BMI were, respectively, z = 9.51, p < .001, z = 7.36, p < .001, z = 3.78, p < .001. Pre- and post-treatment differences were significant when considered separately for patients in group and individual therapy, as well as for patients with BED and those with subthreshold BED, with the exception of a non-significant difference in BMI for the subthreshold BED patient group.

### Assessment of treatment outcome according to the criteria of clinical significance

Table 2 shows differences for the treatment outcome groups with respect to EDE global scores, self-reported binge eating frequency as well as BMI. Since there was only a small number of deteriorated patients (*N* = 3), this group was combined with the unchanged patients (*N* = 44). Based on the EDE-Q global change scores, patients were distributed as follows over the change categories: 62 (44.3%) patients recovered, 31 (22.1%) were reliably improved and 47 (33.6%) patients either remained unchanged or deteriorated.

As to be expected, in the groups classified as recovered or reliably improved, there was a significant decrease in EDE-Q global score with large effect sizes.

Regarding self-reported binge eating frequency (EDE-Q), the results indicate that the recovered and reliably improved groups show a statistically significant decrease with large effect sizes. In the unchanged/deteriorated group the decrease in self-reported binge eating frequency is significant with small to medium effect sizes. Note that in the recovered group the post-treatment mean frequency of binges is near to 0 (*M* = 0.50).

Finally, BMI decreased significantly in the recovered and reliably improved group, although with small effect sizes, while the unchanged/deteriorated group did not show a significant change in BMI.

**Table 2:** Pretreatment and post-treatment means (SD) for outcome groups based on the EDE-Q global score

		Pretreatment scores	Post-treatment scores			
	Outcome variables	M (SD)	M (SD)	Test statistics	*p*	Hedges’s g_av_
Recovered (*n* = 62)	EDE-Q global score	3.51 (0.83)	1.13 (0.55)	t(61) = 20.24	*p* < .001	3.35
	EDE-Q OBE scale scores	5.92 (7.37)	0.50 (1.13)	t(61) = 6.12	*p* < .001	1.02
	BMI	39.02 (5.25)	38.12 (4.95)	t(60) = 3.54	*p <* .001	0.15
Improved (*n* = 31)	EDE-Q global score	4.26 (0.55)	2.96 (0.39)	t(30) = 15.07	*p* < .001	2.63
	EDE-Q OBE scale scores	10.60 (10.18)	1.90 (2.90)	t(29) = 4.96	*p* < .001	1.13
	BMI	38.21 (6.69)	37.28 (6.63)	t(30) = 2.91	*p* = .007	0.14
Unchanged/	EDE-Q global score	3.42 (0.71)	3.26 (0.67)	t(46) = 2.49	*p* = .016	0.22
deteriorated (*n* = 47)	EDE-Q OBE scale scores	6.48 (10.72)	2.54 (3.76)	t(45) = 2.57	*p* = .013	0.48
	BMI	40.39 (6.30)	40.19 (6.01)	t(46) = 0.90	*p* = .371	0.03

Notes: EDE-Q, Eating Disorder Examination Questionnaire; BMI, body mass index; *p* = p value. Hedges’s g_av_ effect size: 0.20 = small; 0.50 = medium; 0.8 = large [67]. Analyses excluding deteriorated patients (*n* = 3) result in equal results in terms of significance. Given occurrences of positive skewness of distributions of difference scores for the above three outcome variables (skewness/SE > 1.96), Wilcoxon tests were computed to confirm all parametric results. Non-parametric test statistics resulted in p-values of comparable size as reported above, with the exception of a clearly smaller p-value for differences in the EDE-Q binge eating frequency in the unchanged/deteriorated group, with z = 2.63, p = .008

### Explaining clinical improvement by personality functioning and personality traits

The associations between clinical improvement and personality functioning and personality traits were investigated by univariate analyses of group differences between outcome groups. In addition, logistic regression analyses were performed to identify predictors of clinical recovery.

#### Univariate analyses of differences between outcome groups

For univariate analyses (ANOVA) of group differences with respect to pretreatment personality functioning (DPI) and personality traits (TCI), significant outcomes are presented in Table 3.

**Table 3:** Significant levels of personality functioning and personality traits for outcome groups based on EDE-Q global scores

	Recovered (*n* = 62)	Improved (*n* = 31)	Unchanged/ deteriorated (*n* = 47)			Test statistics	
	M (SD)	M (SD)	M (SD)		*F †*	η2 ‡	Post hoc t-test §
DPI ‘neurotic’ scale	37.17 (16.41)	45.24 (11.10)	44.34 (15.34)		4.37*	0.06	1 < 2,3*
DPI Resistance	13.40 (5.64)	16.33 (4.32)	15.32 (5.51)		3.60*	0.05	1 < 2*
DPI Dependence	13.82 (6.91)	17.03 (5.53)	17.72 (6.63)		5.42**	0.07	1 < 3*
TCI Harm avoidance	21.86 (6.58)	24.58 (6.04)	24.59 (6.38)		3.22*	0.05	ns

Notes: DPI, Developmental Profile Inventory; TCI, Temperament and Character Inventory; † F obtained by ANOVA; due to missing values, the degrees of freedom for the residuals of the model vary between 136[thus, F(2, 136)] and 137[thus, F(2,137)]; * = *p* ≤ .05; ** = *p* ≤ .01; *** = p ≤ .001. ‡ Cohen’s (1992): eta-squared: 0.02 = small; 0.13 = medium; 0.26 = large. § Post hoc test p-values reported only for significant F-tests. Where Post hoc tests were non-significant ns was reported. Analyses excluding deteriorated patients (n = 3) show equal results in terms of significance. As there were no significant between-group pretreatment differences in age, educational level, BMI or therapy type (group, individual) analyses were not adjusted for any of these potential covariates

Regarding DPI personality functioning, ANOVA showed that for one out of two maladaptive aggregated developmental levels, namely ‘neurotic’ functioning, and two out of nine subsequent developmental levels, namely the maladaptive levels of Resistance and Dependence, there were overall significant differences between outcome groups. Regarding TCI personality traits, one out of seven trait scales, namely Harm avoidance showed significant differences between outcome groups.

To assess pairwise differences between outcome groups post hoc t-tests with Bonferroni corrected *p*-values were performed. Compared to the recovered group, both the improved and unchanged/deteriorated groups had significantly higher scores on the aggregated ‘neurotic’ scale (resp. *p* = .048 and *p* = .044). In addition, the improved group had significantly higher scores on the scale Resistance (*p* = .041) and the unchanged/deteriorated group had significantly higher scores on Dependence (*p* = .007). Effect sizes for these differences were between the boundaries of small to medium. Regarding TCI personality traits significant overall differences were found between groups on Harm avoidance, with the lowest mean scores in the recovered group, however these paired differences did not remain significant after Bonferroni correction for multiple testing.

#### Predictors of recovery

In single logistic regression analyses, mild to moderate depressive disorder (OR = 0.20, 95% CI [0.06, 0.62]), DPI resistance (OR = 0.92, 95% CI [0.87, 0.97]), DPI dependence (OR = 0.92, 95% CI [0.86, 0.98]), the aggregated DPI ‘neurotic’ scale (OR = 0.92, 95% CI [0.86, 0.98]) and TCI harm avoidance (OR = 0.97, 95% CI [0.94, 0.99]) were significant predictors of recovery after treatment. Odds ratios below 1 indicate that for patients with a mild to moderate depressive disorder and higher scores on the aforementioned DPI scales and TCI harm avoidance the odds of being recovered after treatment decrease.

The results of the multiple logistic regression analysis on recovery are presented in Table 4. The predictors entered in the model were mild to moderate depressive disorder, the aggregated DPI ‘neurotic’ scale and TCI Harm avoidance. Significant predictors of recovery in this model were having a mild to moderate depressive disorder (OR = 0.58, 95% CI [0.37, 0.90]) and the aggregated DPI ‘neurotic’ scale (OR = 0.65, 95% CI [0.42, 0.99]). The ROC curve showed a significant AUC of 0.65 (95% CI, 0.55–0.74, *p* = .003), indicating significant predictive value of the multiple logistic regression model.

Note that both DPI levels of Resistance and Dependence are part of the aggregated DPI ‘neurotic’ scale. The aggregated score was preferred as a predictor in the multiple regression model over both separate scales. In a multiple regression model including both separate DPI scales, these predictors were not significant due to their intercorrelation (*r* = .69).

**Table 4:** Multiple logistic regression to predict recovery

Variables	*B*	SE (*B*)	Wald	*p*-value	Exp(*B*)	Exp(*B*) 95% CI
Depressive disorder	-0.55	0.23	5.86	0.015	0.58	0.37, 0.90
DPI ‘neurotic’ scale	-0.44	0.22	3.99	0.046	0.65	0.42, 0.99
TCI Harm avoidance	-0.15	0.22	0.46	0.498	0.86	0.57, 1.32
Constant	-3.05	0.19	2.64	0.104	0.74	

Notes. Nagelkerke *R*^2^ = 0.16, Chi^2^(3) = 17.52, *p* < .001. Predictor variables were standardized prior to the analysis

## Discussion

The aim of the study was to explore the association between levels of personality functioning and specific personality traits and CBT treatment outcome for female obese patients with BED or subthreshold BED. First, we found that CBT was effective in reducing binge eating pathology (as measured with the EDE-Q global score and self-reported binge eating frequency), and in reaching a significant, although small, weight loss. Second, impaired (‘neurotic’) levels of personality functioning, -that is the aggregated ‘neurotic’ scale and the levels of Dependence and Resistance-, as well as higher scores on TCI Harm avoidance were significantly associated with a less favorable outcome after CBT in patients with binge eating. However after correction for multiple testing paired differences for TCI Harm avoidance were not significant.

According to the criteria of clinically significant change in our study a substantial group of patients recovered (44.3%) or improved (22.1%), however a considerable number of patients remained unchanged or deteriorated (33.6%). Recovery in our study also concerned a significant and clinically meaningful reduction in frequency of binge-eating episodes as the mean frequency of binges in the recovered group was decreased after therapy towards near zero (*M* = 0.50 per 28 days), which is even below frequency of binge eating in the general population [51]. Remission rates are a valuable indicator of treatment success in BED. We have aimed to obtain a robust measure of clinically meaningful change by classifying patients in the four different outcome groups based on their EDE-Q global score. Here, you could see the proportion of patients in the ‘recovered’ group (i.e., 44.3%) as an indication of the remission rate in our study. This is supported by the finding that for “self-reported 28-day frequency of objective binge eating episodes” the mean pretreatment score of 5.92 decreased to a mean post-treatment score of 0.5 (near zero).

In addition, recovered patients reached a significant, but small decrease in BMI, although weight loss was not a therapy target in itself. These results are in line with previous studies showing that psychotherapy, mostly CBT, had significant post-treatment effects on binge-eating episodes, with recovery rates ranging from 54 to 63% [4, 12, 14–16, 68]. In addition, these studies reported improved eating disorder psychopathology as reflected in participants’ susceptibility to hunger, cognitive control over eating, and overall concerns about eating, shape and weight when compared to inactive control groups (typically wait-list), whereas effects on weight loss effects was either non-significant or minimal [4, 12, 14–16, 68].

Our second main finding was that recovery after CBT treatment was significantly associated with lower pretreatment scores on ‘neurotic’ personality functioning, (the DPI aggregated ‘neurotic’ scale and the developmental levels Resistance and Dependence). Furthermore, multiple logistic regression analysis, controlling for mild to moderate depressive disorder and TCI Harm avoidance showed that ‘neurotic’ personality functioning was a significant negative predictor of clinically significant change. Where for patients with higher scores on ‘Neurotic’ personality functioning the odds of being recovered after treatment decrease.

Maladaptive ‘neurotic’ personality functioning is characterized by problems with the self (e.g. low self-esteem) and problematic patterns in interpersonal contact, characterized by a lack of autonomy, and by (the avoidance of) conflicts. Our results are in line with previous studies regarding self-esteem in transdiagnostic eating disorders samples, demonstrating that higher scores on self-esteem contributed to a more positive EDE-Q outcome [69, 70]. However, other studies found contradictory results. Vall & Wade [71] report in a meta-analysis that higher self-esteem predicted better outcomes at follow-up, but not at the end of treatment, possibly due to the large differences in the effects reported across the three included studies. Cooper and coworkers report that patients with lower base-line self-esteem achieved a better outcome with CBT-E [72] and in study by Grilo and coworkers [73] self-esteem was not found to be a predictor nor a moderator of CBT outcome. With respect to interpersonal problems, previous studies suggested that a greater extent of interpersonal problems prior to treatment predict more eating disorder pathology at post-treatment in overweight patients with BED [37, 38, 71]. These findings are in line with our results as we found in univariate analyses that higher pretreatment scores on the DPI ‘neurotic’ levels of personality functioning, in particular Resistance and Dependence, were significantly associated with a less favorable outcome after CBT. This was supported by multiple logistic regression analysis, were both mild to moderate depressive disorder and ‘neurotic’ personality functioning were significant negative predictors of clinically significant change.

Regarding personality traits, the results of our study showed significant overall differences on Harm avoidance. In line with our results for DPI personality functioning, the groups with poorer outcomes showed higher pretreatment scores on Harm avoidance, although pairwise differences were not statistically significant after Bonferroni correction. Furthermore, in multiple logistic regression analysis, controlling for mild to moderate depressive disorder and neurotic personality functioning, Harm avoidance was not a significant predictor of clinical recovery. Contradictory to our results, previous studies among patients with eating disorders showed that low ‘Harm avoidance’ predicted a poor clinical outcome in patients with Anorexia Nervosa and Bulimia Nervosa [74] and high ‘Harm avoidance’ predicted favorable clinical changes after a six-month therapy of Brief Adlerian Psychodynamic Psychotherapy for patients with Anorexia Nervosa and Bulimia Nervosa [75]. However, no studies investigated the association between BED treatment outcome and TCI personality traits yet.

As we found that pre-treatment patients with BED had a more maladaptive personality functioning profile than patients with subthreshold BED, we explored BED as potential covariate. Importantly, additional analyses ruled out BED as potential covariate in the analysis of treatment effect and the prediction analysis. However, as the current study was not powered for the comparison of BED and subthreshold BED patients, additional studies, with larger group sizes are necessary. Future research should investigate the suggestion that patients with BED have a more maladaptive personality functioning profile and, subsequently, inform about the relation between those differences and clinical change after CBT.

### Strengths & limitations

A definite strength of this study is the naturalistic treatment setting and the inclusion of a heterogeneous sample including female obese patients with both BED or subthreshold BED and therefore its relevance in clinical practice. Of additional value is the relatively large number of treatment completers. Another potential strength of the study includes the utilization of both statistical and clinical significance methods to explore therapeutic outcomes, reflecting the proportion of patients who not only statistically, but clinically significantly improved, reflecting recovery or improvement after treatment in a meaningful way. In the current study, strong psychometric properties of the EDE-Q global scores warrant the use of the RCI as indicator of non-zero true score change, however McAleavey (2022) presents a discussion of alternative approaches that might be more suited in other contexts [76].

This study is differentiating in its approach studying psychodynamic personality functioning as well as personality traits, which is in line with the AMPD of DSM-5 [2]. Furthermore, the level of personality functioning and core psychodynamic impairments were assessed using the DPI, a dimensional approach that has not yet been used in previous studies of obesity and BED. Moreover, we assessed personality traits using the TCI, a reliable questionnaire for the assessment of relevant personality traits in subjects with and without specific psychopathology [52]. These findings subsequent former study results, showing that obese patients with BED or subthreshold BED presented more maladaptive and less adaptive psychodynamic personality functioning as well impaired personality traits reflected by higher Temperament and Character Inventory (TCI) Harm avoidance and lower Self-directedness as compared to non-bingeing obese controls and normal weight controls [34].

This study has several limitations. First, personality variables and eating disorder symptoms were evaluated by self-report questionnaires, instruments that tend to overestimate the prevalence of psychopathology [46]. Second, due to sample size limitations in the data-analyses we did not distinguish between patients who received group CBT and who received individual CBT. However, there were no differences at baseline in relevant sociodemographic or clinical variables between both treatment groups. Third, the fact that the sample consisted solely of female patients might be considered as a limitation as it may not reflect the associations between personality and outcome of CBT treatment in men. Fourth, the study includes only analyses of treatment completers. Participants dropping out of treatment had significantly higher scores on Harm avoidance and Novelty seeking than patients who completed treatment. Assuming that patients who drop out of treatment are more likely to remain unchanged or deteriorate after treatment, the inclusion of these drop-outs in our analysis would have likely amplified the group differences found for the trait Harm avoidance. Note that even though the amount of drop-out in the current study was low (*n* = 12, 7.1%), drawing conclusions from these findings is tentative. The absolute treatment effect should be interpreted with caution, as the estimates were not based on an intent-to-treat analysis. Fifth, the limited sample size may have reduced the power of the study, which might have affected statistical significance of results, in particular for tests of pairwise differences with Bonferroni correction. Finally, our results reflect short term post-treatment outcomes and may not be generalized to prediction of longer-term follow-up. Larger studies with long-term follow-up are needed to replicate and extend our findings.

### Future recommendations

This study showed associations between treatment outcome and personality characteristics, however association does not imply causation. Therefore, more studies should be performed to inform about the causal relationship between personality functioning and personality traits and treatment outcome in patients with BED or subthreshold BED. Within this frame of reference, measuring treatment outcome according to the concept of clinical significance can be of additional meaningful clinical relevance.

Subsequently, future studies should explore the effects of additional psychotherapeutic interventions targeting maladaptive neurotic personality functioning on BED treatment outcome. Such therapeutic programs or additional interventions should aim to improve feelings of low self-worth and lack of autonomy, as well as enhancing interpersonal skills by decreasing the tendency to engage in passive (aggressive) submission or dependent behavior. As suggested by other research [77], the knowledge generated by such studies may be used in developing more effective, specifically tailored treatments for those patients with BED who fail to improve by the current evidence-based treatment programs. Such a shift in eating disorder research and clinical practice, as suggested by Muzi and coworkers [78] could encourage practitioners to adapt psychotherapy interventions to suit the specific transdiagnostic characteristics (e.g., personality features) of individual patients, to better meet their needs and enhance their therapeutic outcome [78]. A dimensional approach in which the level of psychodynamic personality functioning and personality traits are considered, aimed to optimize indication for personalized psychotherapeutic treatment interventions, help to frame multidimensional and nuanced case conceptualization and constitute the essence of the AMPD in the DSM-5.

## Conclusion

Impaired (‘neurotic’) levels of personality functioning were significantly associated with a less favorable outcome after CBT (treatment) in patients with binge eating. Furthermore, ‘neurotic’ personality functioning in addition to and mild to moderate depressive disorder were identified as negative predictors of clinically significant change. This suggests that for patients with higher scores on ‘neurotic’ personality functioning the odds of being recovered after treatment decrease. This finding suggests that assessment of personality functioning in patients with BED or subthreshold BED could support indication for more specified or augmented care, tailored towards the patients’ personal strengths and vulnerabilities.

## Data Availability

The datasets used and analysed during the current study are available from the corresponding author on reasonable request.
